# Minicells derived from *Escherichia coli* Nissle 1917 for efficient phenylalanine degradation

**DOI:** 10.1128/aem.00944-26

**Published:** 2026-07-21

**Authors:** Xiaoya Tian, Kanghui Ju, Jun Zeng, Xiaona Pang, Jingyu Chen

**Affiliations:** 1 Key Laboratory of Food Bioengineering (China National Light Industry), College of Food Science and Nutritional Engineering, China Agricultural University, Beijing, China; 2 China Agricultural University-Sichuan Advanced Agricultural & Industrial Institute 839103, Chengdu, China; 3 Xinjiang Laboratory of Special Environmental Microbiology, Institute of Microbiology, Xinjiang Academy of Agricultural Sciences 74608, Urumqi, China; 4 Laboratory of Food Quality and Safety, Beijing Key Laboratory of Agricultural Product Detection and Control of Spoilage Organisms and Pesticide Residue, College of Food Science and Engineering, Beijing University of Agriculture 524360, Beijing, China; Michigan State University, East Lansing, Michigan, USA

**Keywords:** minicells, *Escherichia coli *Nissle 1917, phenylalanine degradation, metabolic engineering

## Abstract

Phenylketonuria is an inherited metabolic disorder characterized by impaired phenylalanine (Phe) catabolism and toxic Phe accumulation. Current therapies are limited by poor long-term adherence, high cost, and biosafety concerns associated with existing or emerging approaches. Here, a non-replicative yet metabolically active platform was developed using anucleate minicells derived from *Escherichia coli* Nissle 1917 (EcN). A dual phenylalanine degradation system was introduced to engineer these minicells into synthetic microreactors, designated PCB101. *In vitro*, PCB101 degraded 10 mM Phe within 1.5 h. In a murine hyperphenylalaninemia model, intravenous injection of PCB101 (10^9^ cells/mL) reduced plasma Phe by 87.5%, and oral administration (10^10^ cells/mL) achieved a 66% reduction in a dose-dependent manner. These results demonstrate that EcN-derived minicells provide a controllable and biosafe chassis for efficient Phe degradation both *in vitro* and *in vivo*, supporting their potential applicability in biotherapeutic strategies requiring stable dosing and prevention of microbial proliferation.

**IMPORTANCE:**

Engineered microbial systems are widely applied to perform defined metabolic functions, yet most designs rely on viable and replicating cells, intrinsically coupling functional output to population expansion. This growth-dependent paradigm complicates dose stability and raises biosafety concerns in practical applications. This study demonstrates that anucleate minicells derived from *Escherichia coli* Nissle 1917 retain translational and metabolic activity despite lacking chromosomal DNA and replicative capacity. By incorporating a dual phenylalanine degradation pathway, the engineered minicells efficiently reduced phenylalanine both in vitro and in vivo. This study illustrates that metabolic function can be preserved independently of cell division, providing a controllable microbial platform for applications requiring stable functional output without population expansion. This work expands the conceptual framework for non-replicative microbial systems in applied microbiology.

## INTRODUCTION

Phenylalanine (Phe) is an essential aromatic amino acid that participates in protein synthesis and multiple metabolic processes in humans (1). Under physiological conditions, Phe is converted to tyrosine by phenylalanine hydroxylase (PAH) in a reaction that requires tetrahydrobiopterin (BH_4_) as a cofactor (2). In phenylketonuria (PKU, OMIM #261600) (3), pathogenic variants in PAH or genes involved in BH_4_ biosynthesis impair this conversion, leading to systemic accumulation of Phe (4). In untreated individuals, plasma Phe concentrations often exceed 1,200 μM, resulting in the formation of neurotoxic metabolites that disrupt brain development and function (5, 6). If not adequately managed, PKU causes irreversible neurological impairment, including intellectual disability, seizures, and behavioral abnormalities.

Despite decades of clinical experience, PKU remains a disease with limited long-term therapeutic options. Dietary restriction of Phe has been the cornerstone of clinical management for decades (5, 7, 8). However, this approach requires lifelong avoidance of natural protein intake and dependence on specialized medical foods, which frequently leads to poor adherence and nutritional imbalance, particularly in adolescents and adults (9). These limitations have driven continued efforts to develop alternative strategies that directly reduce systemic Phe burden rather than relying solely on dietary control (10). Enzyme-based approaches have emerged as one such strategy. Enzyme replacement therapy using pegylated phenylalanine ammonia lyase (PAL) (11) can effectively lower plasma Phe levels, but its clinical application is constrained by extremely high cost, immune-mediated hypersensitivity reactions, and the need for repeated administration due to limited durability (12–14). In parallel, gene therapy has been explored as a potential curative approach (15). However, this strategy remains at an early research stage and faces substantial ethical, technical, and economic barriers (7, 16). Vector delivery efficiency, long-term safety, and patient-specific variability currently preclude its broad clinical implementation, and even successful cases would likely remain prohibitively expensive as individualized treatments (17–19).

In recent years, increasing attention has been directed toward live biotherapeutic strategies that aim to degrade Phe directly within the gastrointestinal tract. Engineered probiotic strains, particularly *Escherichia coli* Nissle 1917 (EcN), have been modified to express PAL in order to reduce intestinal Phe absorption (20, 21). Preclinical studies have demonstrated clear biochemical activity *in vitro* and in animal models, highlighting the conceptual promise of this approach. However, translation to clinical efficacy has been limited, with reported trials showing insufficient improvement in disease outcomes (22). These shortcomings reflect fundamental challenges associated with live bacterial systems, including uncontrolled microbial replication, variability in *in vivo* metabolic activity, difficulty in maintaining stable dosing, and concerns regarding biosafety and biological dissemination (23, 24). These limitations highlight a central bottleneck in the development of live biotherapeutics for PKU: the need to retain metabolic activity while eliminating uncontrolled proliferation and long-term colonization. An ideal system would provide predictable and confined biochemical function without introducing risks associated with microbial expansion or horizontal gene transfer. Addressing this gap requires a delivery chassis that decouples metabolic capability from bacterial replication (25).

Minicells offer such a possibility. Historically, minicells were used as simplified systems to study bacterial division, protein synthesis, and bacteriophage infection (26, 27). More recently, their nanoscale size, intrinsic biocompatibility, and low immunogenicity have prompted exploration of minicells as delivery vehicles for drugs and genetic cargo, particularly in oncology and gene engineering applications (28, 29). Minicells are nanoscale anucleate derivatives of rod-shaped bacteria generated by abnormal polar division (30, 31). They are 100–400 nm in diameter and produced when the Min system (encoded by the *minB* gene cluster: *minC*, *minD*, and *minE*) is disrupted (29). Loss of Min system function leads to mislocalized Z-ring assembly, producing daughter cells that lack chromosomal DNA (32, 33). Although minicells cannot divide or sustain long-term growth, they retain intact membranes, ribosomes, RNA, enzymes, and plasmid DNA inherited from the parental cell (34). As a result, minicells preserve short-term metabolic activity and protein synthesis capacity while eliminating risks associated with replication, gut colonization, and ecological persistence.

This unique physiological state enables a functional separation between biological activity and genetic inheritance. Minicells maintain sufficient ATP production and translational capacity to support defined biochemical reactions over limited time windows. In addition, asymmetric segregation of ribosomes and plasmids during minicell formation can produce controlled heterogeneity in protein expression, allowing reproducible tuning of metabolic output across a population (35, 36). Compared with conventional nanoparticle systems, minicells offer several advantages (37, 38). Many synthetic nanoparticles exhibit poor biodegradability, drug leakage, or manufacturing challenges at scale (39). In contrast, minicells are biologically derived, readily loaded with proteins or nucleic acids, and naturally cleared after functional exhaustion. These properties have enabled their use as carriers for therapeutic agents and gene delivery tools, including experimental applications involving CRISPR–Cas systems for *in vivo* gene modulation (40). Importantly, these prior studies demonstrate that minicells can function as confined and controllable biological units without long-term persistence.

This study aims to provide a novel way by using EcN modified minicells as a biological chassis for Phe degradation. EcN was selected as the parental strain due to its well-established probiotic safety profile and prior use in metabolic engineering studies. By converting EcN into minicells, the system is designed to retain enzymatic degradation capacity while eliminating bacterial replication. Within these minicells, a phenylalanine-responsive degradation system was constructed to enable efficient Phe catabolism. This design aims to combine the metabolic effectiveness of live biotherapeutics with the dose control and biosafety typically associated with non-replicative delivery systems, thereby addressing key translational barriers in PKU-related live biotherapeutic research.

## RESULTS

### Construction, purification, and characterization of minicells in EcN

**Fig 1:**
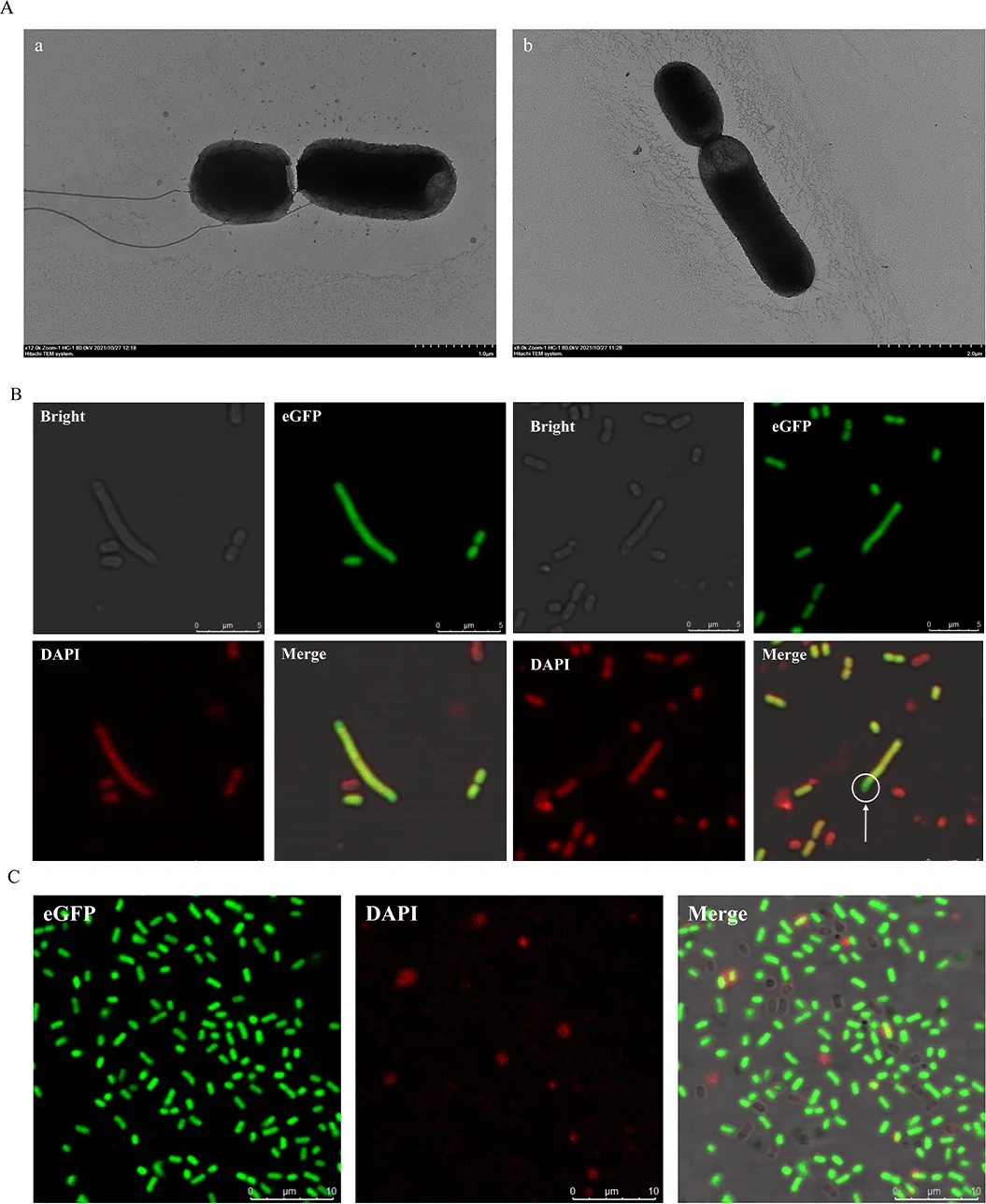
Morphological characterization and purification of EcN-derived minicells. (**A**) Transmission electron microscopy (TEM) images (a and b) of an EcN-Δ*minCDE* minicell showing abnormal division. Scale bars, 1.00 μm. TEM analysis was performed using a Hitachi HT7800 microscope (80.0 kV acceleration voltage, ×12,000 magnification). (**B**) Local and global field-of-view CLSM images of EcN-Δ*minCDE*. Scale bars, 5.0 μm. Local and global field-of-view images showing eGFP fluorescence (green, indicating metabolic activity) and absence of DAPI staining (red, confirming anucleation). “Bright” represents bright-field images, and “merge” denotes the overlay of eGFP, DAPI, and bright-field images. Arrows in the merge subfigures point to target minicells, which exhibit distinct green eGFP fluorescence (validating metabolic activity) but no red DAPI staining (confirming their anucleate nature). (**C**) Global field-of-view CLSM images of the isolated minicell.

Minicells were generated from EcN by scarless deletion of the *minCDE* operon using CRISPR-Cpf1. Loss of the Min system redirected cell division from the midcell to polar regions, leading to the formation of anucleate minicells. Transmission electron microscopy revealed abnormal polar division in the engineered strain (EcN-Δ*minCDE*), with spherical or short rod-shaped minicells budding from bacterial poles (Fig. 1A). To verify the absence of chromosomal DNA while preserving metabolic activity, EcN-Δ*minCDE* was transformed with an eGFP-expressing plasmid and stained with DAPI. Confocal microscopy showed strong eGFP fluorescence in polar minicells, whereas DAPI signals were absent, confirming their anucleate nature and retained translational activity (Fig. 1B). A two-step purification method combining antibiotic treatment and gradient centrifugation is developed to isolate minicells from parent cells (Fig. S1). The purity of minicells was confirmed by the spread plate method and microscopic examination (Fig. 1C). The percentage of red parent cells labeled with DAPI alone was significantly reduced in the overlay of eGFP excitation and labeling. Fluorescence-based quantification showed that DAPI-negative eGFP-positive minicells accounted for more than 90% of total fluorescent particles after purification, indicating effective removal of parental-cell contaminants.

### Engineering the Phe metabolic pathway into minicells

**Fig 2:**
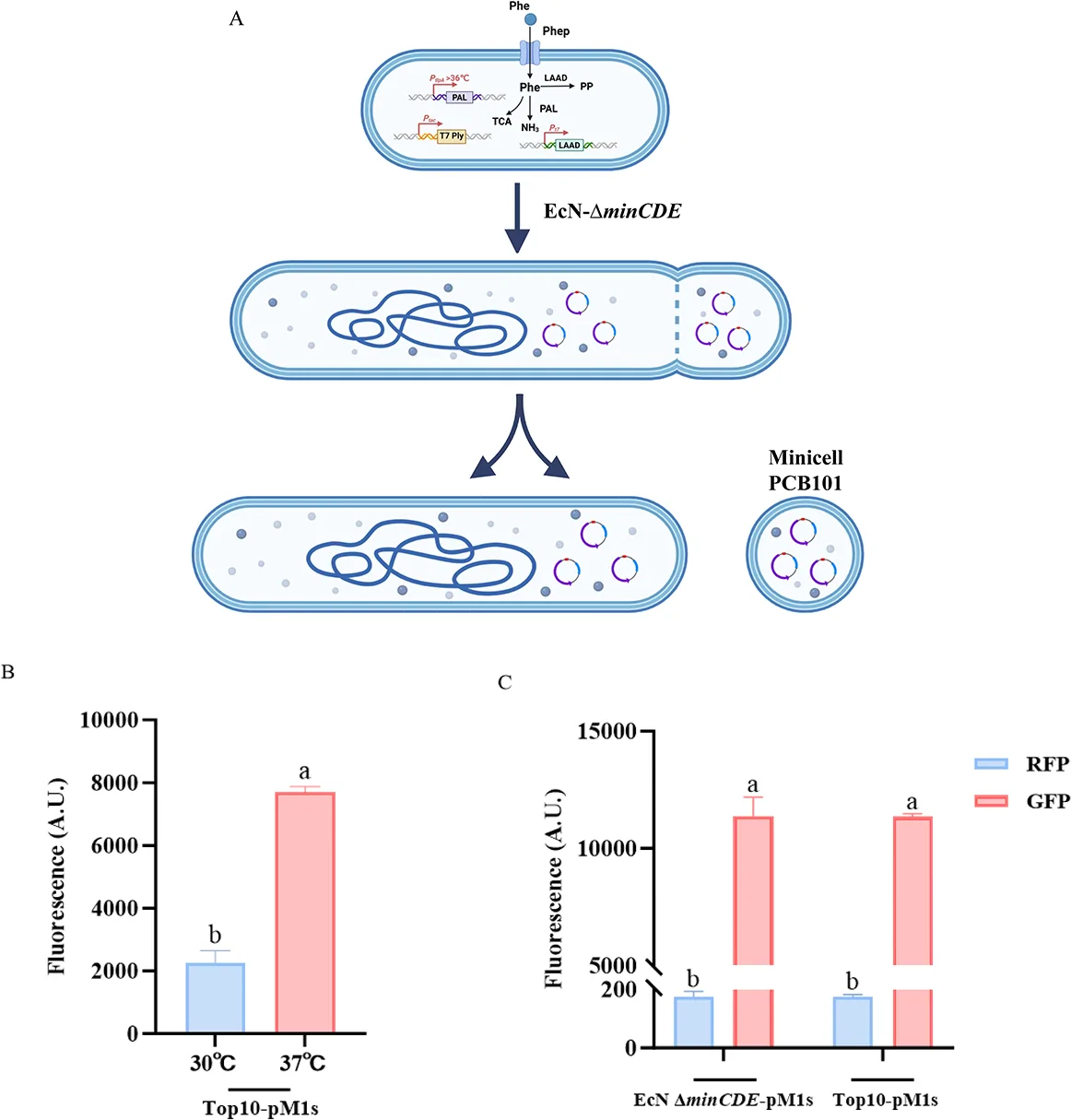
Construction of PCB101 and optimization of the temperature-sensitive promoter *pTlpA*. (**A**) Schematic diagram of PCB101 construction. (i) Temperature-sensitive promoter *pTlpA* drives expression of PAL and high-affinity Phe transporter PheP; (ii) IPTG-inducible promoter *pTac* regulates T7 RNA polymerase, which activates T7 promoter-mediated expression of LAAD. Plasmids used: pM1s3AsR_TS-C7 (carrying PAL/PheP and *pTlpA*), pACYDuet (carrying LAAD and T7 promoter), and pGEX-4T-2-T7 RNA pol-kan (carrying T7 RNA polymerase and *pTac*). (**B**) Absolute fluorescence quantification of *pTlpA*-driven eGFP in *E. coli* Top10 at 30°C (suppressed state) and 37°C (activated state) (mean ± SD, *n* = 3 independent experiments, *P* < 0.001, *t*-tests). Fluorescence was measured using a microplate reader (excitation: 488 nm, emission: 509 nm). (**C**) Comparative analysis of promoter strength: fluorescence expression comparison between GFP (green) driven by *pTlpA* and RFP (red) driven by the constitutive promoter *pJ23101* in *E. coli* Top10 and EcN-Δ*minCDE* at 37°C. Quantitative analysis of fluorescence intensity confirming *pTlpA* (37°C) exhibited 2.3-fold higher expression than *pJ23101* (mean ± SD, *n* = 3 independent experiments, *P* < 0.0001, one-way ANOVA).

To construct a minicell-based Phe degradation system, PAL, LAAD, and PheP were introduced into EcN-derived minicells to establish two complementary catabolic routes (Fig. 2A). This engineering strategy was adapted from earlier studies of engineered *E. coli* live biotherapeutics for PKU (22), which established PAL-dependent phenylalanine degradation as a feasible metabolic intervention, but was modified here to accommodate the physiological constraints of a non-replicative minicell platform. Because minicells lack chromosomal DNA, all heterologous expression modules were implemented using plasmid-based systems. To improve plasmid compatibility and expression stability, the native cryptic plasmid pMut1 was removed and replaced with the reconstructed vector pM1s3AsR_TS-C7. PAL expression was placed under control of the temperature-responsive promoter *pTlpA*, while the LAAD pathway was regulated independently through the T7 expression module. The performance of *pTlpA* was evaluated using a fluorescence reporter assay. At 37°C, *pTlpA*-driven GFP expression was readily detected in engineered minicells (Fig. 2B). Quantitative fluorescence analysis showed that *pTlpA* exhibited significantly stronger expression activity than the constitutive promoter *pJ23101* under induction conditions (Fig. 2C). These results confirmed that the temperature-responsive regulatory system provided robust heterologous expression capacity for subsequent metabolic pathway implementation.

### *In vitro* degradation efficiency of engineered minicells for Phe metabolism

**Fig 3:**
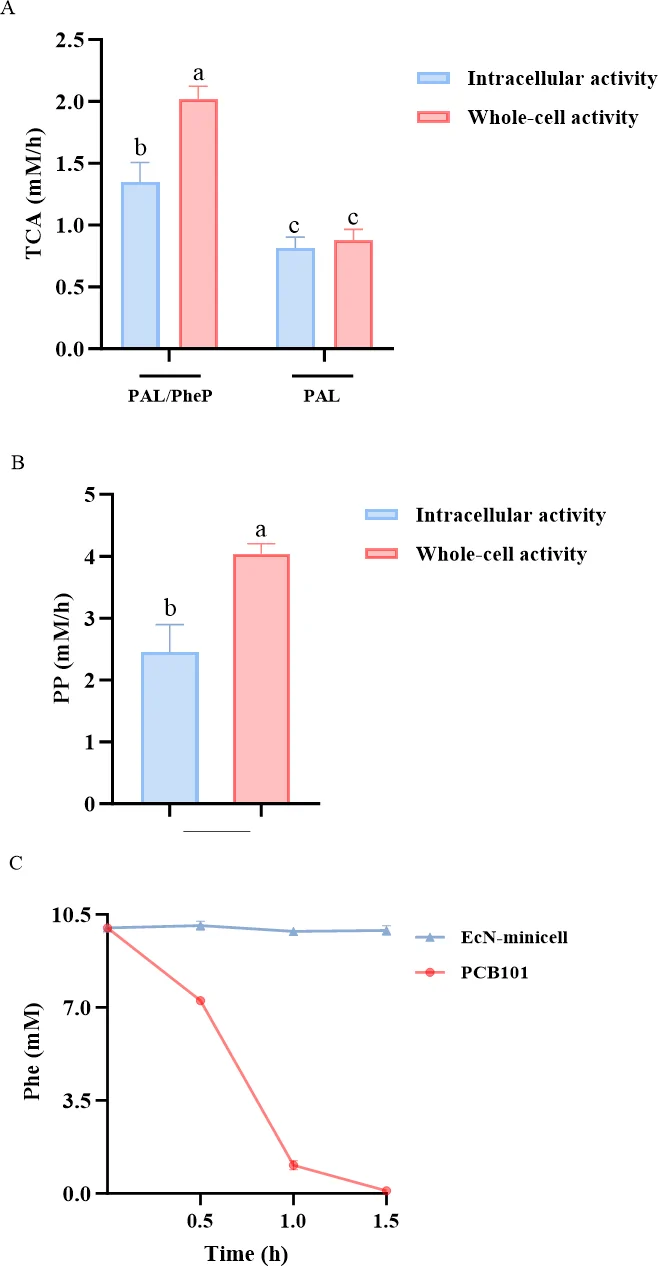
Biodegradation activity and Phe degradation of PCB101 *in vitro*. (**A**) Intracellular and whole-cell Phe degradation activity of PAL and PAL degradation activity after co-expression with PheP. Intracellular activity and whole-cell activity were measured by HPLC. Co-expression of PheP enhanced whole-cell degradation rate from 0.8 mM/h (PAL alone) to 2.0 mM/h (mean ± SD, *n* = 3, *P* < 0.01). (**B**) Intracellular and whole-cell Phe degradation activity of LAAD, with a maximum whole-cell rate of 4.0 mM/h (mean ± SD, *n* = 3). (**C**) Whole-cell Phe degradation capacity of PCB101. Purified minicells (2 × 10^10^ cells/mL) were incubated with 10 mM Phe (100 mM Tris-HCl, pH = 8) at 37°C. PCB101 degraded Phe almost completely within 1.5 h, while EcN minicells (negative control) showed no significant degradation (mean ± SD, *n* = 3, *P* < 0.001). Samples were taken at regular intervals to detect residual Phe by HPLC. HPLC analysis was performed using a Jasco system equipped with an Agilent ZORBAX SB-C18 column (250 mm × 4.6 mm, 5 µm) at a detection wavelength of 260 nm.

To distinguish intrinsic catalytic activity from transport-dependent degradation within intact minicells, Phe degradation was evaluated using both minicell lysates and intact purified minicell preparations. Lysate-based assays were used to assess enzymatic activity independent of membrane transport, whereas intact-particle assays were used to evaluate integrated substrate uptake and conversion within the preserved minicell architecture. Expression of PAL alone in purified minicells supported measurable Phe degradation, with an intact-particle degradation rate of 0.8 mM/h at a normalized particle concentration of 2 × 10^10^ cells/mL (Fig. 3A). Co-expression of PAL and the Phe transporter PheP significantly increased degradation activity to 2.0 mM/h, representing an approximately 2.5-fold increase relative to PAL alone (Fig. 3A). This result indicated that Phe transport was a limiting factor for PAL-dependent conversion in intact minicells. Minicells expressing LAAD showed a higher degradation rate of 4.0 mM/h under aerobic conditions (Fig. 3B), consistent with efficient oxidative deamination activity. The intracellular activity profiles were consistent with intact-particle measurements, supporting retention of catalytic competence following minicell purification. Protein induction was optimal when overnight cultures were subcultured to OD_600_ = 0.4 and induced with 0.2 mM IPTG for 4 h. Under these conditions, intact purified PCB101 minicells degraded 10 mM Phe completely within 1.5 h (Fig. 3C). In contrast, purified minicells derived from wild-type EcN showed no detectable Phe degradation under identical conditions. These results demonstrate that engineered minicells retained sufficient transport and metabolic functionality to support dual-pathway Phe degradation after purification. The final engineered minicell platform was designated PCB101 (Fig. 2A).

### Engineering a storage system for minicells

**Fig 4:**
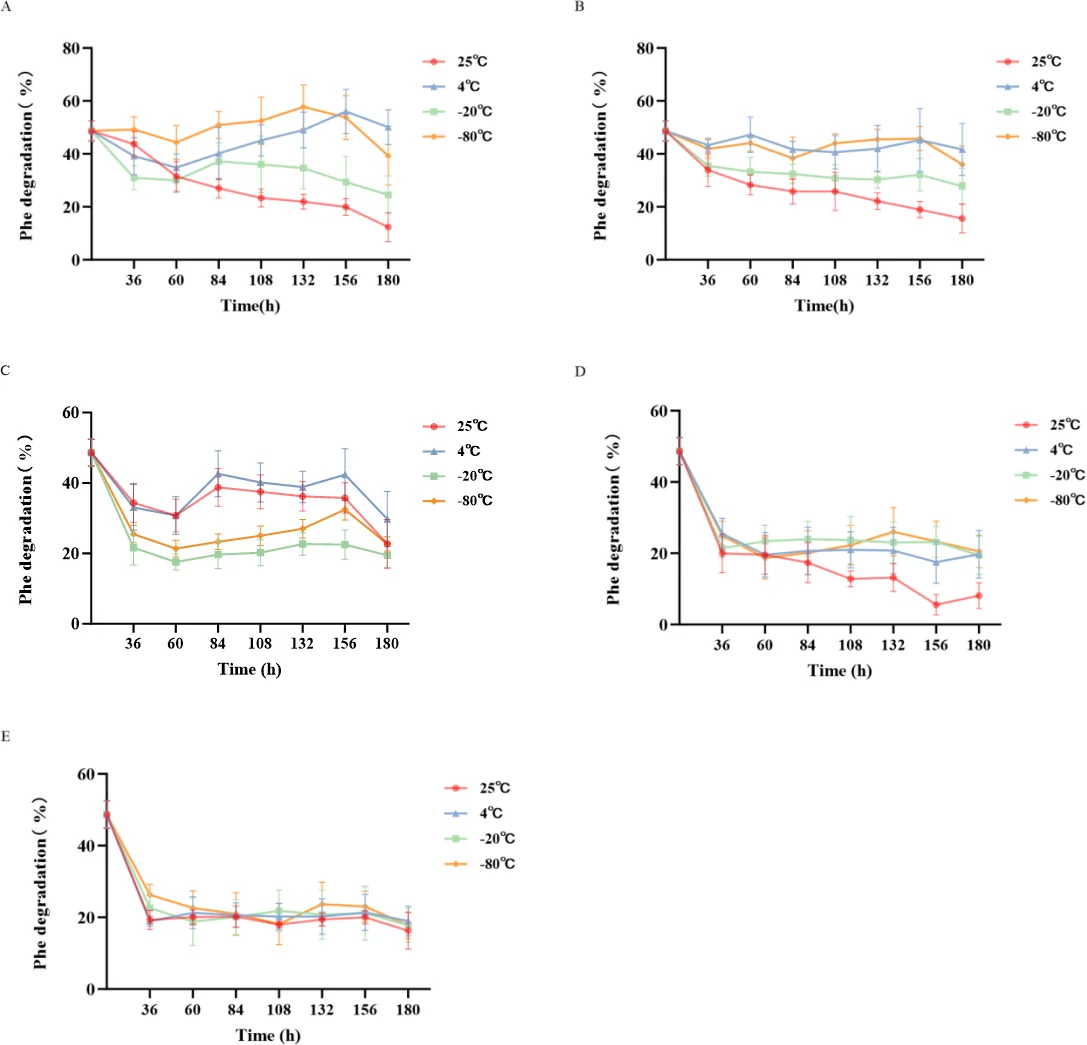
Residual Phe degradation activity of PCB101 under different storage conditions. (**A–E**) Phe degradation activity of PCB101 stored in preservation systems (**A**) 15% glycerol, (**B**) M9 salts + 15% glycerol, (**C**) 30% glycerol, (**D**) M9 salts + 30% glycerol, and (**E**) PBS. Samples were stored at 25°C (room temperature), 4°C, −20°C, and −80°C for 7 days, and Phe disappearance was observed over time in different stored conditions. Residual degradation activity was detected by HPLC (*n* = 3 independent experiments). Only initial rates were determined from samples (0–2 days), as either temperature or preservation systems led to a non-linear decrease of Phe degradation activity.

The stability and bioactivity of minicells are crucial for their potential applications in biotechnology, such as drug delivery and vaccine development. Several widely used probiotic protection systems (including 15% glycerol, 30% glycerol, M9 salts + 15% glycerol, M9 salts + 30% glycerol, and phosphate-buffered saline [PBS]) were tested to identify a suitable protective agent for maintaining the activity of PCB101 to facilitate further development and application. The effects of PCB101 under short-term storage conditions were evaluated in these preservation systems (Fig. 4). Among all formulations tested, 15% glycerol consistently provided the most stable preservation of PCB101 bioactivity, maintaining more than 80% of initial degradation activity after 7 days at 4℃ (Fig. 4A). Storage at −20℃ and −80℃ showed comparable preservation, whereas activity declined more rapidly at room temperature. The addition of M9 salts to 15% glycerol did not produce measurable improvement in activity retention and resulted in slightly lower residual activity under several storage conditions (Fig. 4B), suggesting that nutrient salt supplementation did not provide additional stabilization under the conditions tested. PCB101 stored in 30% glycerol or M9 salts supplemented with 30% glycerol showed comparable but more variable preservation performance (Fig. 4C and D). In contrast, storage in PBS led to substantially faster activity loss across all temperatures (Fig. 4E). Taken together, these results identify 15% glycerol at 4℃ as the most practical preservation condition, providing robust short-term stability while avoiding unnecessary formulation complexity. Notably, the stable bioactivity of PCB101 at −20℃ and −80℃ in the 15% glycerol system provides the possibility for the long-term storage of minicells, which is critical for batch production, transportation, and off-the-shelf application of minicell-based therapeutics.

### *In vivo* Phe degradation by engineered minicells

**Fig 5:**
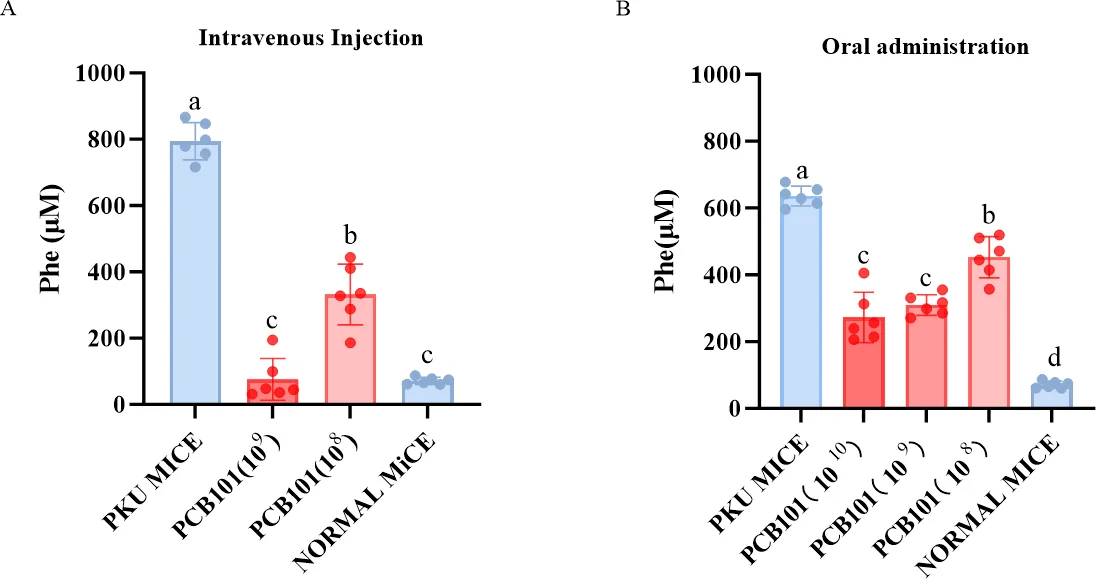
*In vivo* Phe degradation by PCB101 in PKU mice. (**A**) Phe degradation effect in mice after intravenous injection of PCB101 (*n* = 6 per group). PKU model mice were injected via tail vein with PCB101 at two doses (10^9^ and 10^8^ cells/mL). After experimental testing, intravenous injection of 10^10^ cells/mL of PCB101 into the tail of mice would affect the normal activity function of the mice. Plasma Phe levels were measured by HPLC 1.5 h post-administration. The high-dose group achieved 87.5% Phe reduction (from approximately 800 to 100 µM), while the low-dose group achieved 62.5% reduction. (**B**) Phe degradation effect in mice after oral administration of PCB101 (*n* = 6 per group). PKU model mice were gavaged with PCB101 at three doses (10^10^, 10, and 10^8^ cells/mL). The 10^10^ cells/mL dose achieved 66% Phe reduction, with a significant dose-dependent effect. The PKU model was established by intraperitoneal injection of p-CP (300 mg/kg) + Phe (100 mg/kg) daily for 7 days, with plasma Phe stabilized at ~800 µM. The control group (normal mice) received PBS. Different lowercase letters (a, b, c, and d) above the bars indicate significant differences among groups (mean ± SD, *P* < 0.001, one-way ANOVA).

To evaluate the *in vivo* activity of PCB101, mice were administered L-Phe and p-chlorophenylalanine (p-CP) weekly in 6-day cycles. Mouse plasma was then extracted, and the resulting serum was analyzed by high-performance liquid chromatography (HPLC) to measure plasma Phe levels. Using a sustained modeling method, the average Phe level in mouse plasma was stabilized at approximately 600 µM. In subsequent experiments, the amount of Phe supplementation for mice was increased to create a model more similar to the gene-knockout PKU animal model, ultimately resulting in a stable plasma Phe level of approximately 800 µM. During the experimental process, it was observed that the oral gavage of Phe led to a more significant increase in mouse plasma Phe levels compared to intraperitoneal injection, indicating that oral administration mimics the normal absorption and metabolic processes in the body. This strategy allows the agent to first reach the gastrointestinal tract and then be absorbed through the digestive system, potentially facilitating more uniform distribution within the body. In contrast, with intraperitoneal injection, agents may come into contact with abdominal cavity enzymes and undergo degradation. Two delivery modes were tested in this study: oral administration and intravenous injection. Consistent with this rationale, the study innovatively injected PCB101 at two titers into the tail veins of PKU mice. Both treatment groups showed significant therapeutic results compared to the control group (Fig. 5A). Among them, the high-concentration minicell injection group (10^9^ cells/mL) achieved an approximate degradation rate of 87.5%, while the low-concentration group also reached a degradation rate of 62.5%. Patients with PKU have plasma Phe levels of approximately 1 mM, suggesting that PCB101 has high potential for future therapeutic success. Notably, during the experiment, mice in the high-concentration administration group exhibited more active states and physical signs more similar to normal mice, with significant symptom relief, in contrast to mice in the PKU control group and other groups, which displayed more lethargic activity.

PCB101 effectively reduced the absorption of Phe from the gastrointestinal tract into the bloodstream through oral dosing. The results showed that 10^10^ cells/mL of PCB101 could degrade approximately 66% of serum Phe levels (Fig. 5B). Furthermore, three oral dosage gradients were established, and the results showed a significant dose-dependent effect on the final degradation of Phe, suggesting that the dosage can be effectively adjusted to control the Phe degradation effect.

### Inflammatory response assessment of engineered minicell PCB101

**Fig 6:**
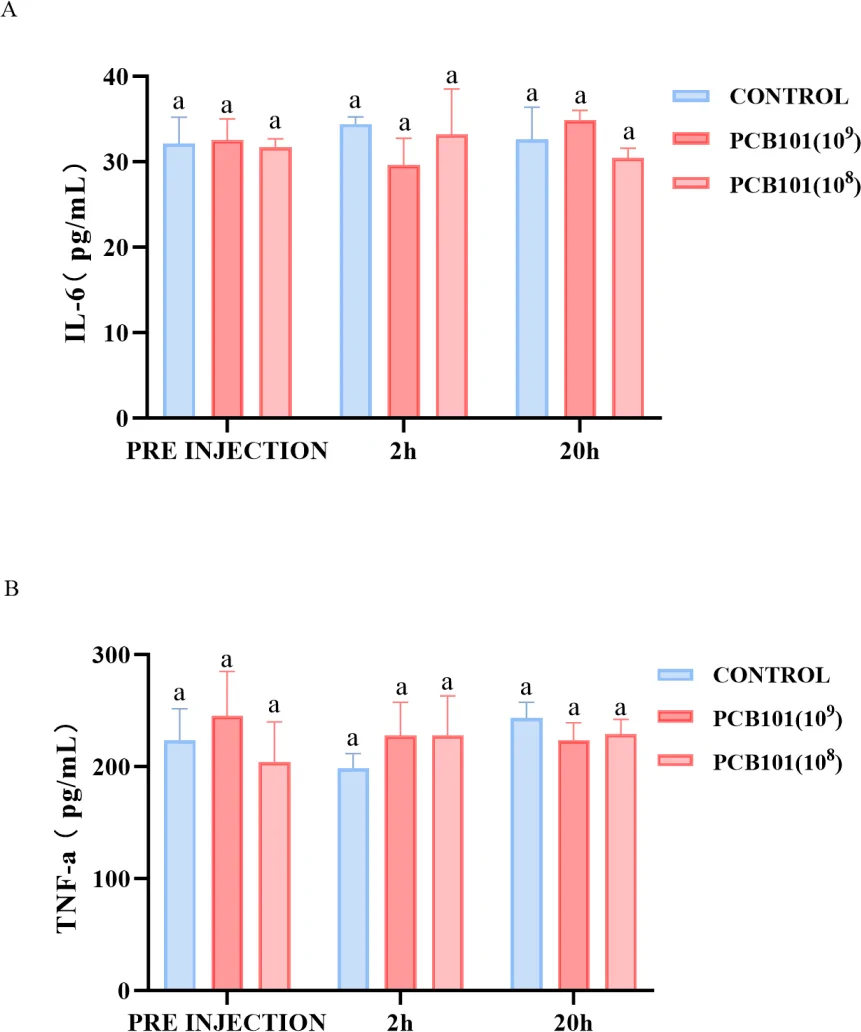
Levels of inflammatory factors induced by intravenous injection of PCB101 in mice. (**A**) IL-6 and (**B**) TNF-α levels in serum of normal mice (*n* = 6 per group) before injection (0 h), 2 h (acute phase), and 20 h (steady phase) post-intravenous administration of PCB101 (10^9^ or 10^10^ cells/mL). No significant elevation of cytokines was observed compared to the PBS control group. EcN-derived minicells showed no significant TNF-α induction, confirming low immunogenicity. Cytokine levels were measured using commercial ELISA kits (Mouse TNF-α/IL-6 ELISA Kit) with a microplate reader (*λ*_max_ = 450 nm). The lowercase letter ”a” above the bars indicates significant differences among the groups (mean ± SD, *P* > 0.05, two-way ANOVA).

This study measured the levels of TNF-α and IL-6 in normal mice following a single intravenous injection of two titers (Fig. 6). The results showed that intravenous administration of two titers of EcN minicells did not significantly increase the levels of these two inflammatory factors during the acute inflammatory phase (2 h after injection) compared to the PBS placebo group and preinjection levels. There was also no significant variation in inflammatory factor levels during the steady phase (20 h after injection). Additionally, when administering intravenous doses to PKU mice, the experimental process found that even after consecutive 2-day high-dose minicell injections, there were no abnormal changes in body temperature, food intake, hematological indicators, or other external signs (Fig. 6A and B), demonstrating the safety of *in vivo* minicell administration.

## DISCUSSION

**Fig 7:**
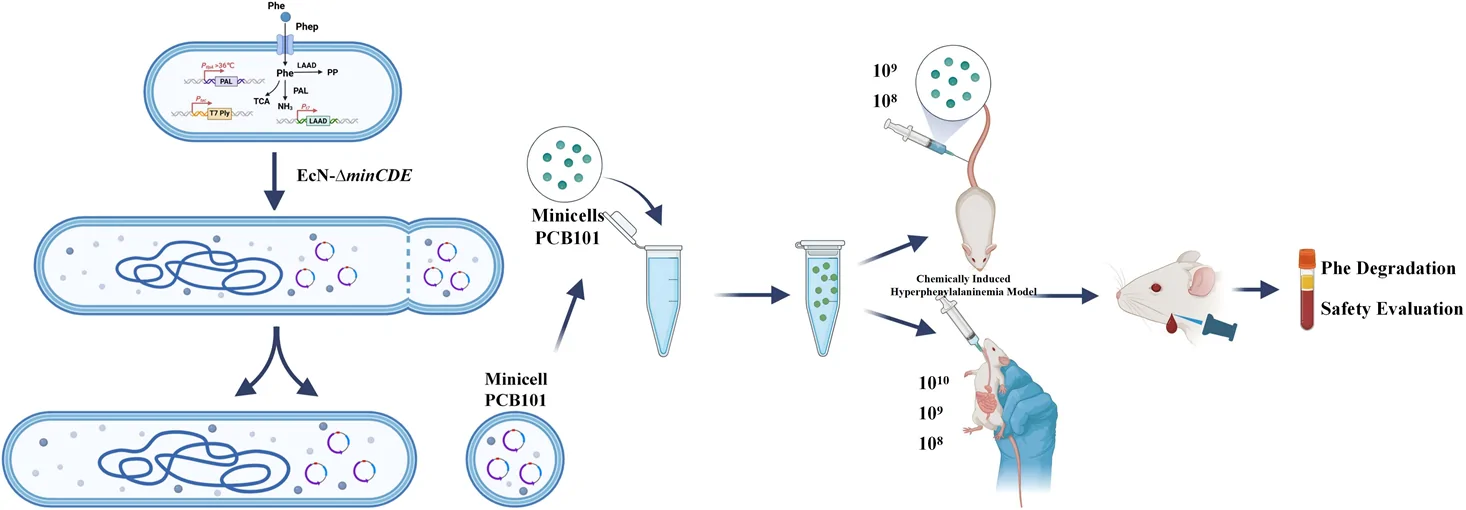
Workflow diagram showing EcN-delta minCDE bacterial engineering to produce minicells PCB101 expressing PAL, PheP and LAAD for Phe degradation, tested via injection and oral administration in a chemical-induced hyperphenylalaninemia mouse model.

Minicells have recently attracted increasing attention as biologically derived delivery platforms because they retain essential intracellular machinery while lacking chromosomal DNA and proliferative capacity. Early studies primarily explored their utility as passive carriers for therapeutic cargo delivery, particularly in oncology and nucleic acid transport. More recent work has suggested that minicells may also retain sufficient metabolic competence to support active biochemical functions (41, 42). The present study extends this emerging concept by demonstrating that EcN-derived minicells can be engineered as metabolically functional units for *in vivo* phenylalanine degradation (Fig. 7). A central feature of the minicell platform is the physical separation of metabolic activity from cellular replication. The design logic underlying PCB101 is rooted in the structural and metabolic consequences of the anucleate state. In replication-competent bacteria, a substantial fraction of intracellular resources is continuously allocated toward DNA replication, chromosome maintenance, and cell division. By contrast, minicells generated through disruption of the Min system are structurally committed to a non-replicative configuration while retaining translational machinery, membrane transport systems, and preassembled metabolic components inherited from the parental cell. This configuration redistributes intracellular resources away from growth-associated processes and toward maintenance of existing biochemical functions. Such resource reallocation may contribute to the relatively stable short-term metabolic output observed in this study and distinguishes minicells from stationary-phase bacteria, which retain replicative potential but undergo broad metabolic downregulation in response to nutrient limitation.

Previous work by Isabella et al. (22) established that dual-pathway Phe degradation in replicative *E. coli* enhances *in vivo* robustness by enabling the organism to adapt to fluctuating oxygen levels and substrate availability. The present study employs a comparable complementary pathway architecture, with PAL/PheP operating under anaerobic conditions and LAAD extending degradation capacity in aerobic environments, but the underlying functional rationale differs substantively from prior replicative systems. In a replicative organism, pathway redundancy supports organismal fitness and persistence. In minicells, which lack adaptive regulation and replication, the same configuration functions to stabilize metabolic output within a closed system across heterogeneous physiological conditions. Temperature-sensitive promoter control further reflects this system-level orientation. By coupling pathway expression to physiological temperature, the platform achieves autonomous activation under *in vivo* conditions without dependence on exogenous chemical inducers. This strategy is particularly well suited to non-replicative systems where precise temporal control compensates for the absence of adaptive cellular responses and represents a broader design principle for context-aware regulation in acellular synthetic biology platforms. *In vivo* evaluation demonstrated that PCB101 retains metabolic functionality following both oral and intravenous administration. Intravenous injection at 10⁹ cells/mL reduced plasma Phe by 87.5%, while oral administration at 10^10^ cells/mL achieved a 66% reduction in a dose-dependent manner. These results support the premise that non-replicative, metabolically active systems can function within complex biological environments. The absence of detectable inflammatory or toxic responses, even at high doses, is consistent with the improved safety profile expected of anucleate, non-replicating carriers.

However, several limitations of the current data must be acknowledged. The murine model employed relies on exogenous Phe and p-chlorophenylalanine loading rather than a genetically defined hyperphenylalaninemia background, such as PAH-knockout mice. As a result, the contribution of minicell-mediated degradation cannot be fully disentangled from host metabolic variability and compensatory regulation. The observed reductions in plasma Phe are consistent with active minicell function, but more controlled models and extended time-course measurements will be needed to establish causal attribution. These limitations do not invalidate the proof-of-concept findings, but they define the scope of conclusions that can currently be drawn. Additional constraints are inherent to the non-replicative architecture itself. Minicells possess finite functional lifespans, with enzymatic and metabolic activity declining over time due to the absence of biosynthetic renewal. Reduced regulatory capacity also limits dynamic responsiveness to environmental fluctuations. These trade-offs, namely, the tension between predictability and adaptability and between safety and functional durability, are fundamental to the design logic of acellular platforms and will require ongoing attention as the system is developed further. Strategies from protein engineering and formulation science, including approaches used to extend enzyme half-life in nanoparticle delivery systems, may be adapted to prolong minicell metabolic activity and partially address these limitations.

Beyond the specific application to Phe metabolism, this work introduces a generalizable design framework for non-replicative metabolic function delivery. Minicells combine the biological functionality of living cells, the safety of non-replicating systems, and the modularity of synthetic carriers. By retaining ribosomes, cofactors, and membrane transporters, they support sustained enzymatic activity in ways, while avoiding the genetic containment challenges of live bacterial therapeutics. PKU serves here as a representative benchmark for metabolite accumulation disorders, but the same platform logic is applicable to other conditions requiring controlled *in vivo* enzymatic activity.

In summary, this study advances the role of probiotic-derived minicells from passive carriers to metabolically active biological modules capable of executing complex biochemical functions *in vivo*. The broader contribution lies in establishing biological containment through cellular structure as a viable alternative to genetic containment strategies, and in demonstrating that metabolic functions can be engineered as deployable, time-limited system properties. Future work should focus on validating efficacy in more stringent disease models, extending functional lifespan through protein and formulation engineering, and exploring the generalizability of this platform to other small-molecule-related therapeutic applications.

## MATERIALS AND METHODS

### Bacterial strains, plasmids, and culture conditions

All bacterial strains and plasmids used in this study are listed in Table 1. EcN served as the parental strain, while *E. coli* DH5α competent cells (Shanghai Wedi Biotechnology Co., Ltd.) were used for recombinant plasmid construction. Unless otherwise specified, strains were cultured in super optimal broth medium (20.00 g/L tryptone, 5.00 g/L yeast extract, 0.50 g/L NaCl, 0.19 g/L KCl, 0.95 g/L MgCl_2_, and 3.60 g/L glucose) at 37℃ with constant shaking. When necessary, the medium was supplemented with 100 µg/mL of ampicillin, 50 µg/mL of kanamycin, 50 µg/mL of streptomycin, or 15 µg/mL of chloramphenicol to support plasmid propagation.

**TABLE 1:** Strains constructed in this study

Strain or plasmid	Description	Source
Strains		
*E. coli* DH5α	F⁻ φ80 *lac* ZΔM15 Δ(*lacZYA-argF*) U169 *endA1 recA1 hsdR17*(rk⁻, mk^+^) *supE44* λ⁻ thi⁻ *gyrA96 relA1 phoA*	Lab stock
*E. coli* (Nissle 1917)	Wild type	Lab stock
EcN-Δ*minCDE*	Nissle 1917 without cryptic plasmid pMut1, Δ*minCDE*	This study
PCB101	Minicells derived from EcN-Δ*minCDE*, containing M1s3AsR_TS-C7-*stla-phep*, pGEX-4T-2-T7 RNA pol-Kan, pACYDuet-LAAD, Kan^R^, Amp^R^, Cm^R^	This study
Plasmids		
pEcCpf1	*rep101*, *pCas*-FnCpf1, *pAraB*-Red, *pRhaB*-crRNA-pMB1, *rhaS*, *rhaR*, *sacB*, Kan^R^	(43)
pcrEG	*PMB1*, *aadA*, J23119-N23 insert *pVeg*-sfGFP, Sm^R^	(43)
pcrEG-*minCDE*	pcrEG, containing crRNA*_minCDE_* and homologous arms for *minCDE* deletion, Sm^R^	This study
pM1s3AsR_TS-C7	pMUT1 backbone, ColE1 ori, constitutive promoter *pJ23101* driving mRFP1, temperature-sensitive promoter *pTlpA* driving GFP, Amp^R^	Lab stock
pM1s3AsR_TS-C7-*stlA*	pM1s3AsR_TS-C7 containing the *stlA* gene from *Photorhabdus luminescens* TT01, Amp^R^	This study
pM1s3AsR_TS-C7-*stlA-phep*	pM1s3AsR_TS-C7-*stla* containing the *phep* gene from *E. coli* str. K-12 substr. MG1655, Amp^R^	This study
pGEX-4T-2	ColE1 ori, *lacIq*, *pTac*, Amp^R^	Lab stock
pGEX-4T-2-T7 RNA ply-Kan	pGEX-4T-2, containing T7 RNA polymerase, Kan^R^	This study
pACYCDuet-1	*p15A* ori, *cat*, *lacI*, dual *pT7*, Cm^R^	Lab stock
pACYCDuet-LAAD	pACYCDuet-1, containing the *pma* gene from *Shewanella putrefaciens*	This study

### Construction of minicell-producing strains

The *minCDE* operon was deleted from EcN using CRISPR-Cpf1-mediated genome editing, as previously described (43), generating the minicell-producing strain EcN-Δ*minCDE*. Successful deletion was confirmed by PCR and Sanger sequencing (Beijing Tsingke Biotech Co., Ltd.). The wild-type and mutant strains were maintained under identical growth conditions. All gene sequences used in this study are listed in the supplemental material (Table S1). All the primers used for gene editing are listed in Table S1.

### Minicell isolation and purification

Minicell purification was performed by combining antibiotic treatment and gradient centrifugation (Fig. S1). Cultures were harvested at mid-log phase and then centrifuged at 2,000 × *g* for 10 min to remove 90% of the parent cells (27). The supernatant was centrifuged at 10,000 × *g* for 10 min to pellet the minicells. The collected minicells were resuspended and incubated with ceftriaxone sodium (100 µg/mL) at 37℃ for 1 h, followed by centrifugation at 400 × *g* for 5 min to remove cellular debris and dead cells. Subsequent centrifugation and washing steps were performed to remove cellular debris. Final purification was achieved by filtration through a 0.22 µm membrane. Minicell purity was quantified by fluorescence microscopy following dual staining with DAPI and plasmid-derived eGFP fluorescence. Purified minicell suspensions were imaged using confocal laser scanning microscopy. Ten randomly selected fields from three independent preparations were analyzed. Particles exhibiting eGFP fluorescence but lacking detectable DAPI signal were classified as minicells, whereas particles positive for both eGFP and DAPI were classified as residual parental cells. Minicell purity was calculated as the percentage of DAPI-negative eGFP-positive particles relative to total eGFP-positive particles. Minicell morphology and division patterns were examined by transmission electron microscopy and scanning electron microscopy.

### Evaluation of the thermoregulated promoter *pTlpA*

The temperature-sensitive promoter *pTlpA* was evaluated by comparing its transcriptional strength with that of the constitutive promoter *pJ23101* using fluorescent reporter genes. In the reporter plasmid pM1s3AsR_TS-07, GFP expression was controlled by *pTlpA*, while RFP expression was driven by *pJ23101*, as previously described (44). Reporter constructs were transformed into *E. coli* Top10 and EcN-Δ*minCDE*. Cultures were grown to mid-log phase at 37℃, harvested, washed, and resuspended in PBS. Fluorescence intensities of GFP (Ex/Em: 488/509 nm) and RFP (Ex/Em: 532/588 nm) were measured using a fluorescence microplate reader. Absolute fluorescence values were used for promoter strength comparison.

### Plasmid construction

The PheP protein-encoding gene from *E. coli* str. K-12 substr. MG1655 (GenBank accession number NC_000913.3) and the *stlA* gene expressing PAL from *Photorhabdus luminescens* TT01 (GenBank accession number WP_225190393.1) were carefully inserted into the pM1s3AsR_TS-C7 vector (45) using the *BamH*I restriction site in homologous recombination. Furthermore, the *pma* gene expressing LAAD from *Shewanella putrefaciens* was constructed into the pACYCDuet vector (GenBank accession number U35383.1). The above gene sequences are listed in Table S2. EcN-*ΔminCDE* was electroporated with these plasmids (Bio-Rad; 1.8 kV pulse, 1 mm gap cuvette). To resolve ampicillin resistance conflicts between pGEX-4T-2-T7 RNA polymerase and pM1s3AsR_TS-C7, the ampicillin resistance gene in pGEX-4T-2-T7 was replaced with a kanamycin resistance gene via seamless cloning, as described previously (46) with optimized primer design for higher recombination efficiency. Positive transformants (Amp/Cm/Kan) were verified by sequencing and glycerol-preserved. To construct plasmids, the gene fragment and linearized plasmid backbone were amplified by the corresponding primers through a PCR and ligated through one-step cloning using the Uniclone One Step Seamless Cloning Kit (Genesand Biotech Co., Ltd, Beijing, China). The DNA sequences of all primers used for constructing the Phe degradation pathway are detailed in Table S3.

### Optimization of induction conditions and analysis of Phe degradation

Optimal induction conditions (IPTG concentration: 0.01–1 mM, induction time: 4–7 h, and growth stage: OD = 0.1–0.5) were optimized *in vitro*. Purified minicells were resuspended in 10 mM Phe buffer (100 mM Tris-HCl, pH = 8) for induction. The OD_600_ was set to the same value for each treatment group. After 1.5 h, supernatants were collected and filtered (0.22 µm) for HPLC analysis.

Phe, PP, TCA, and other metabolites were quantified using HPLC (LC-2050; Shimadzu, Japan), with an Agilent ZORBAX SB-C18 column (250 mm × 4.6 mm, 5 µm) and an ultraviolet (UV) detector. The mobile phase consisted of 4% (vol/vol) acetonitrile and was isocratically eluted (injection volume: 20 µL, column temperature: 40℃). Phe quantification was performed at a constant flow rate of 1 mL/min for 12 min, based on peak areas obtained at 260 nm and a retention time of 6 min for Phe (46).

### Minicell-based enzymatic activity assay

For intracellular enzyme activity assays, minicells were disrupted using an ultrasonic cell disruptor (300 W, 5 min). Cell debris was removed by centrifugation at 12,000 × *g* for 10 min, and the supernatant was used as the crude enzyme extract. Enzyme activity was determined by monitoring Phe consumption and product formation (TCA for PAL, PP for LAAD) using HPLC, as described previously (47). One unit of enzyme activity was defined as the amount of enzyme required to degrade 1 µmol of Phe per minute at 37℃. For whole-particle activity measurements, purified minicells obtained after sequential antibiotic treatment, differential centrifugation, and filtration were directly resuspended in reaction buffer without lysis. Residual parental-cell contamination was assessed independently as described above.

### Storage conditions and activity evaluation of PCB101

Purified PCB101 samples were stored in PBS, 15% glycerol, 30% glycerol, M9 salts supplemented with 15% glycerol, and M9 salts supplemented with 30% glycerol. Samples were stored at 25℃, 4℃, −20℃, or −80℃. At designated time points, aliquots were withdrawn and assayed for residual Phe degradation activity as described in “Optimization of induction conditions and analysis of Phe degradation,” above. Activity retention was normalized to the initial activity measured immediately after purification.

### Modeling and serum analysis of PKU in mice

C57 BL/6J male wild-type mice (10–12 weeks) in this experiment were purchased from Beijing Sinogenetic Biotechnology Co., Ltd. All experimental animals were kept at a temperature of 22℃ ± 2℃, a relative humidity of 50%–60%, and a 12 h light–dark cycle. Animals had free access to standard experimental animal chow and water. All mice were housed under specific pathogen-free conditions and randomly assigned to experimental groups (*n* = 6 per group). Mice were acclimatized for 7 days before experimentation.

A chemically induced hyperphenylalaninemia model was established by intraperitoneal injection of p-CP (300 mg/kg) and L-Phe (100 mg/kg) daily at 16:00 for 7 days. One hour after p-CP/Phe injection, mice received an oral gavage of L-Phe (200 mg/kg). On day 7, approximately 100 µL of whole blood was collected via submandibular bleeding into heparinized tubes. Blood samples were centrifuged at 3,000 × *g* for 10 min at 4℃ to separate serum. Serum proteins were precipitated by adding an equal volume of 0.59 M perchloric acid, vortexed for 30 s, and incubated at room temperature for 15 min. The mixture was centrifuged at 12,000 × *g* for 5 min, and the supernatant was filtered through a 0.22 µm membrane for HPLC analysis.

### *In vivo* different administration methods of PCB101

#### Intravenous injection experimental group

Mice were randomly divided into three groups (*n* = 6 per group): (i) normal control, intraperitoneal injection of 200 µL sterile water daily for 6 days; (ii) model control, intraperitoneal injection of p-CP (300 mg/kg) and Phe (100 mg/kg) daily for 6 days, followed by oral gavage of Phe (200 mg/kg) 30 min later; (iii) treatment group, same as the model control for 4 days, then intravenous injection of PCB101 minicells (10^8^ or 10^9^ cells/mL) via the tail vein 1.5 h after Phe gavage on days 5 and 6. On day 6, mice were euthanized 1.5 h after the final injection, and blood samples were collected for serum Phe analysis.

#### Oral administration experimental group

Mice were randomly divided into five groups (*n* = 6 per group): (i) normal control, intraperitoneal injection of 200 µL sterile water daily for 6 days; (ii) model control, intraperitoneal injection of p-CP (300 mg/kg) daily for 6 days, followed by oral gavage of Phe (50 mg/kg) 30 min later; (iii–v) treatment groups, same as the model control, but oral gavage of PCB101 minicells (10^8^, 10^9^, or 10^10^ cells/mL) mixed with Phe (50 mg/kg) 30 min after p-CP injection. On day 6, mice were euthanized 1.5 h after the final gavage, and blood samples were collected for serum Phe analysis.

### Determination of inflammatory cytokines TNF-α and IL-6

Mice were intravenously injected with PCB101 minicells (10^9^ or 10^10^ cells/mL) or PBS (control). Blood samples (70 µL) were collected via submandibular bleeding at 0, 2, and 20 h post-injection. Serum was separated by centrifugation at 3,000 × *g* for 10 min, and the levels of TNF-α and IL-6 were determined using Mouse TNF-α and IL-6 ELISA Kits (Beijing Solarbio Science & Technology Co., Ltd., China) according to the manufacturer’s instructions. The absorbance was measured at 450 nm using a microplate reader (Allsheng FlexA-200), and cytokine concentrations were calculated from standard curves using four-parameter logistic regression.

### Statistical analysis

Descriptive statistics and hypothesis testing were performed using GraphPad Prism 10. All experiments were independently repeated at least three times, and results are presented as mean ± SD. Differences among groups were analyzed using one-way repeated measures ANOVA or t tests. Post hoc multiple comparisons were conducted using Tukey’s multiple range test with a significance level of *P* < 0.05.

## SUPPLEMENTAL MATERIAL

Supplemental material aem.00944-26-s0001.docx Fig. S1; Tables S1 to S3.

ASM does not own the copyrights to Supplemental Material that may be linked to, or accessed through, an article. The authors have granted ASM a non-exclusive, world-wide license to publish the Supplemental Material files. Please contact the corresponding author directly for reuse.
