# Supplementary material for: Minicells derived from Escherichia coli Nissle 1917 for efficient phenylalanine degradation
Source: Appl Environ Microbiol. 2026 Jul 21;92(8):e00944-26. doi: 10.1128/aem.00944-26 (PMC13488319; doi:10.1128/aem.00944-26)
Supplement: Supplementary file 1 [file aem.00944-26-s0001.docx]

***Supplementary materials***

**Minicells Derived from *Escherichia coli* Nissle 1917 for Efficient Phenylalanine Degradation**

**Xiaoya Tian^1, 2^, Kanghui Ju****^1^, Jun Zeng ^3^, Xiaona Pang^4^, Jingyu Chen^1, 2,^** *

*Affiliations:*

^1^ *Key Laboratory of Food Bioengineering (China National Light Industry), College of Food Science and Nutritional Engineering, China Agricultural University, Beijing 100083, China*

^2^ *China Agricultural University-Sichuan Advanced Agricultural & Industrial Institute, Chengdu 611430, China*

^3^ *Institute of Microbiology, Xinjiang Academy of Agricultural Sciences / Xinjiang Laboratory of Special Environmental Microbiology, Urumqi, Xinjiang 830091, China*

^4^ *Laboratory of Food Quality and Safety, Beijing Key Laboratory of Agricultural Product Detection and Control of Spoilage Organisms and Pesticide Residue, College of Food Science and Engineering, Beijing University of Agriculture, Beijing 102206, China*

* Corresponding author: Jingyu Chen

Address: 17 Qinghua East Rd., Beijing 100083, China

E-mail: [chenjy@cau.edu.cn](mailto:chenjy@cau.edu.cn)

Phone: +86-10-62737966

Fig. S1 Schematic of the two-step purification workflow: (i) Differential centrifugation (2000×*g* for 10 min, then 10,000×*g* for 10 min) to enrich minicells; (ii) Incubation with ceftriaxone sodium (100 µg/mL, 37℃ for 1 h) to eliminate residual parent bacteria; (iii) Filtration through a 0.22 µm membrane to remove endotoxins and debris.

Table S1 Primers used to construct minicell-producing strains

| **Gene** | **Primer** | **Sequence (5’-3’)** |
| --- | --- | --- |
| ***minCDE*** | *minCDE*-1F | gcaaagatgcgcaacttaaagccaatttctactgttgtagatcgtctctgaa |
|  | *minCDE*-2R | ccgcattatctgccgcagttgcgatctacaacagtagaaattatttaaagttcttag |
|  | *minCDE-*pcrEG-2F | tcgagttcatgtgcagctcc |
|  | *minCDE-*pcrEG-1R | gtagggataacagggtaatagatctaag |
|  | *minCDE-*pcrEG-VF | taaccgtattaccgcctttg |
|  | *minCDE-*pcrEG-VR | gtgtgagttaaagttgtactcgag |
|  | Δ*minCDE-*VF | gtgacttgcctcaatataatccag |
|  | Δ*minCDE-*VR | caagaatactcgccgcttt |

Table S2 Genes sequences used to construct strains of Phe metabolic pathway

| **Gene** | **Sequence (5’-3’)** |
| --- | --- |
| ***minCDE*** | atgtcacaatcgccaattgagttaaaaggcagtagttttaccctatcggtcattcatttacatgattcacgaccggaggtaatccgtcaggctttacaggaaaaggtagatcaagcgcccgctttcctgaaaaacgcccctgttgtgattaacgttgcaacgttacctaacggtgctaactggaaagatctccaacaggctgtcacttcagcgggtctgcgtattgttggcatcagtggttgtcaggatgaacgtcaaaaacgtgcgatagcacgggctggtttgccattactgagcgaaggtaaaggtcagaaaatggcaccagagccggttgttagtccgccggaaaatgttcctaccaagacacgaatcatcaacacgcctgtccgttctggccaacagatttatgcccgtaattgtgatttgattgttatcagcagtgtcagtgctggagctgaattaattgccgacggcaatattcatatctatggaatgatgcgcggtcgtgcgctagcaggcgcatcaggtgatgcgcagtgccagattttttgcacgcatctgggtgctgaactagtctctatcgcagggcagtactggttgagtgatcaaatccccagtgattattttggtcaagctgcgcgtttgcatttgctggataacgcattaactatacaacctttaaattaagcccttttgacaaggaatccatttcatggcacgcattattgttgttacatcgggtaaagggggcgttggcaagaccacatcaagcgcggctatcgcaaccggcttggcccaaaaaggtaaaaaaaccgttgttatcgattttgatatcggactacggaatctagacttgattatgggttgtgaacgccgggtagtttacgattttgttaatgtgattcaaggtgacgccacattgaaccaggcattaataaaagataagcgcacggataatttgtatattctgcccgcttctcagacccgtgacaaagacgctctgaccaaagagggtgtcgaaaagattttaaacgatcttggcgaaatgaattttgagtttgtcgtgtgtgactcacctgccggtatcgaaagcggtgcgttgatggcactgtattttgctgacgaagctgtcatcacgaccaaccctgaagtttcttcagttcgtgactcagaccgtatcctcgggatattgtcatccaagtcacgccgagctgagaatggtcaggaaccaattaaagaacatctgctgttgactcgctacaacccagggcgtgttaatcgcggcgatatgcttagcatggaagatgtccttgatattctgaggataccactggttggcgttattccagaagatcagtctgtattgcgcgcctcgaaccaaggcgagcctgtgattctggacaaagagtcagatgccggtaaagcttatgacgataccgttgaccgtttgctaggagaagaacgtcccttccgcttcattgaagaagagaagaagggcttcctgaaacgcctttttgggggataaaccatggctttgttagacttttttctgtcccgcaaaaaaccgacagccaatatagccaaggaacggctgcaaattatcgtcgctgagcgtcgtcgtggggacagtgagccccattatttgcctgacttgaaacgcgatattttggcggttatttgtaaatatattcagattgaccctgaaatgctgcatgtgcaatttgagcagaaaggggatgatatttcggtacttgaacttaacgtgacattaccggaaacggaagaaacgcctaaatga |
| *stlA* | ATGAAAGCGAAAGATGTTCAGCCGACCATCATCATTAACAAAAACGGCCTGATCAGCCTGGAAGATATCTACGATATCGCGATTAAACAGAAAAAAGTTGAAATCAGCGCAGAAATCACCGAACTGCTGACCCACGGCCGTGAAAAACTGGAAGAAAAACTGAACAGCGGCGAAGTTATTTATGGTATCAACACCGGCTTCGGTGGCAACGCGAACCTGGTGGTTCCGTTCGAAAAAATCAGTGAACACCAGCAGAACCTGCTGACCTTTCTGAGCGCTGGCACCGGCGATTATATGTCCAAACCGTGCATCAAAGCTTCCCAGTTCACCATCCTGCTGAGCGTTTGCAAAGGTTGGTCCGCGACCCGTCCGATCGTTGCACAGGCGATTGTTGACCACATCAACAACGATATTGTGCCGCTGGTTCCGCGTTACGGTAGCGTTGGCGCGTCTGGTGATCTGATCCCGCTGTCTTACATTGCCCGCGCGCTGTGCGGCATCGGTAAAGTTTATTACATGGGTGCAGAAATCGACGCGGCAGAAGCGATCAAACGTGCAGGCCTGACCCCGCTGTCCCTGGAAGCCAAAGAAGGTCTGGCACTGATCAACGGTACCCGTGTTATGTCTGGTATCAGCGCCATCACCGTGATCAAACTGGAAAAACTGTTCAAAGCGTCCATCTCTGCAATTGCACTGGCGGTTGAAGCTCTGCTGGCCTCTCACGAACATTACGATGCGCGTATCCAGCAAGTTAAAAACCACCCAGGCCAGAAAGCCGTAGCATCCGCACTGCGCAACCTCCTGGCGGGCTCTACCCAGATCAACCTGCTGACCGGTATCAAAGAACAGGCGAACAAAGCTTGCCGTCACCAGGAAGTGACTCGCCTGAACGACACCCTCCAGGAAGTTTATAGCATTCGCTGCGCTCCGCAGATCCTGGGCATCGTTCCGGAATCCCTGGCTACCGCTCGTAAAATCCTGGAACGTGAAGTGATTAGCGCGAACGACAACCCGCTGATCGACCCGGAAAACGGTGATGTTCTGCATGGTGGCAACTTCATGGGCCAATACGTAGCGCGTACCATGGATGCGCTGAAACTGGATATCGCACTGATCGCTAACCACCTGCACGCGATCGTGGCCCTGATGATGGACAACCGTTTTTCTCGTGGCCTGCCGAACAGCCTGAGCCCGACTCCGGGCATGTACCAGGGCTTCAAAGGCGTGCAGCTGTCTCAGACCGCGCTGGTTGCGGCCATTCGTCACGATTGCGCGGCGAGCGGCATCCACACCTTAGCAACTGAACAGTACAACCAGGATATCGTTTCTCTGGGTCTGCACGCGGCACAGGACGTGCTGGAAATGGAACAGAAACTGCGTAACATCGTGGCCATGACCATCCTGGTTGTTTGCCAGGCGATCCATCTGCGTGGTAACATCTCTGAAATTGCACCGGAAACTGCGAAATTCTATCACGCTGTTCGTGAAATCTCTAGCCCGCTGATCACCGACCGCGCGCTGGATGAAGATATCACCCGTATCGCGGACGCGATCATCAACGACCAGCTGCCGCTGCCGGAAATCATGCTGGAAGAA |
| *pma* | CATATGAACATCAGCCGTCGTAAATTGCTGCTGGGTGTTGGTGCTGCGGGTGTACTGGCAGGTGGTGCTGCTCTTGTTCCGATGGTTCGTCGTGACGGTAAATTCGTTGAAGCGAAATCTCGTGCTAGCTTCGTGGAAGGTACTCAAGGTGCACTGCCAAAAGAAGCAGACGTTGTTATCATCGGTGCAGGTATCCAGGGTATCATGACCGCGATCAACCTGGCTGAACGTGGTATGTCTGTTACCATCTTGGAGAAAGGTCAGATCGCAGGTGAACAGAGCGGTCGTGCTTACTCTCAGATCATCAGCTACCAGACCTCTCCAGAAATCTTTCCGCTGCACCACTACGGTAAGATCTTGTGGCGTGGTATGAACGAGAAGATCGGTGCGGATACCAGCTACCGTACTCAGGGTCGTGTTGAAGCACTGGCGGACGAGAAAGCGCTGGACAAAGCGCAGGCATGGATCAAGACCGCTAAAGAAGCGGCTGGTTTCGACACTCCGCTGAACACTCGTATCATCAAAGGTGAAGAACTGTCTAACCGTCTGGTAGGTGCGCAGACTCCGTGGACCGTTGCTGCGTTCGAAGAAGACTCTGGTTCCGTAGATCCGGAAACCGGTACTCCGGCTCTGGCTCGTTACGCGAAACAGATCGGTGTTAAGATCTACACCAACTGTGCGGTTCGTGGTATCGAAACTGCTGGTGGTAAGATCTCTGACGTTGTTTCTGAGAAAGGTGCGATCAAAACCTCTCAGGTAGTTCTGGCGGGTGGTATCTGGTCTCGTCTGTTCATGGGTAACATGGGTATCGACATCCCAACTCTGAACGTTTACCTGTCTCAGCAGCGTGTTTCTGGTGTACCGGGTGCTCCGCGTGGTAACGTTCATCTGCCGAACGGTATCCACTTCCGTGAACAGGCAGACGGTACTTACGCGGTTGCTCCGCGTATCTTCACCAGCTCCATCGTGAAAGACAGCTTTCTGCTGGGTCCGAAATTCATGCACCTGCTGGGTGGTGGCGAATTGCCACTGGAATTTAGCATCGGTGAAGACCTGTTCAACAGCTTCAAGATGCCGACCAGCTGGAACCTGGACGAGAAGACTCCATTCGAACAGTTCCGTGTTGCAACCGCTACTCAGAACACTCAGCACCTGGATGCTGTGTTCCAGCGTATGAAGACCGAATTTCCGGTGTTCGAGAAATCTGAAGTGGTTGAACGTTGGGGTGCAGTTGTTTCTCCGACCTTCGACGAACTGCCGATCATCTCTGAAGTTAAAGAATATCCGGGTCTGGTTATCAACACCGCTACCGTTTGGGGTATGACCGAAGGTCCGGCAGCTGGTGAAGTAACCGCAGACATCGTTATGGGTAAGAAACCGGTTATCGATCCGACTCCGTTCTCTCTGGACCGTTTCAAGAAGCTCGAG |
| *phep* | GTGAAAAACGCGTCAACCGTATCGGAAGATACTGCGTCGAATCAAGAGCCGACGCTTCATCGCGGATTACATAACCGTCATATTCAACTGATTGCGTTGGGTGGCGCAATTGGTACTGGTCTGTTTCTTGGCATTGGCCCGGCGATTCAGATGGCGGGTCCGGCTGTATTGCTGGGCTACGGCGTCGCCGGGATCATCGCTTTCCTGATTATGCGCCAGCTTGGCGAAATGGTGGTTGAGGAGCCGGTATCCGGTTCATTTGCCCACTTTGCCTATAAATACTGGGGACCGTTTGCGGGCTTCCTCTCTGGCTGGAACTACTGGGTAATGTTCGTGCTGGTGGGAATGGCAGAGCTGACCGCTGCGGGCATCTATATGCAGTACTGGTTCCCGGATGTTCCAACGTGGATTTGGGCTGCCGCCTTCTTTATTATCATCAACGCCGTTAACCTGGTGAACGTGCGCTTATATGGCGAAACCGAGTTCTGGTTTGCGTTGATTAAAGTGCTGGCAATCATCGGTATGATCGGCTTTGGCCTGTGGCTGCTGTTTTCTGGTCACGGCGGCGAGAAAGCCAGTATCGACAACCTCTGGCGCTACGGTGGTTTCTTCGCCACCGGCTGGAATGGGCTGATTTTGTCGCTGGCGGTAATTATGTTCTCCTTCGGCGGTCTGGAGCTGATTGGGATTACTGCCGCTGAAGCGCGCGATCCGGAAAAAAGCATTCCAAAAGCGGTAAATCAGGTGGTGTATCGCATCCTGCTGTTTTACATCGGTTCACTGGTGGTTTTACTGGCGCTCTATCCGTGGGTGGAAGTGAAATCCAACAGTAGCCCGTTTGTGATGATTTTCCATAATCTCGACAGCAACGTGGTAGCTTCTGCGCTGAACTTCGTCATTCTGGTAGCATCGCTGTCAGTGTATAACAGCGGGGTTTACTCTAACAGCCGCATGCTGTTTGGCCTTTCTGTGCAGGGTAATGCGCCGAAGTTTTTGACTCGCGTCAGCCGTCGCGGTGTGCCGATTAACTCGCTGATGCTTTCCGGAGCGATCACTTCGCTGGTGGTGTTAATCAACTATCTGCTGCCGCAAAAAGCGTTTGGTCTGCTGATGGCGCTGGTGGTAGCAACGCTGCTGTTGAACTGGATTATGATCTGTCTGGCGCATCTGCGTTTTCGTGCAGCGATGCGACGTCAGGGGCGTGAAACACAGTTTAAGGCGCTGCTCTATCCGTTCGGCAACTATCTCTGCATTGCCTTCCTCGGCATGATTTTGCTGCTGATGTGCACGATGGATGATATGCGCTTGTCAGCGATCCTGCTGCCGGTGTGGATTGTATTCCTGTTTATGGCATTTAAAACGCTGCGTCGGAAATAA |
| T7 RNA ply | atgaacacgattaacatcgctaagaacgacttctctgacatcgaactggctgctatcccgttcaacactctggctgaccattacggtgagcgtttagctcgcgaacagttggcccttgagcatgagtcttacgagatgggtgaagcacgcttccgcaagatgtttgagcgtcaacttaaagctggtgaggttgcggataacgctgccgccaagcctctcatcactaccctactccctaagatgattgcacgcatcaacgactggtttgaggaagtgaaagctaagcgcggcaagcgcccgacagccttccagttcctgcaagaaatcaagccggaagccgtagcgtacatcaccattaagaccactctggcttgcctaaccagtgctgacaatacaaccgttcaggctgtagcaagcgcaatcggtcgggccattgaggacgaggctcgcttcggtcgtatccgtgaccttgaagctaagcacttcaagaaaaacgttgaggaacaactcaacaagcgcgtagggcacgtctacaagaaagcatttatgcaagttgtcgaggctgacatgctctctaagggtctactcggtggcgaggcgtggtcttcgtggcataaggaagactctattcatgtaggagtacgctgcatcgagatgctcattgagtcaaccggaatggttagcttacaccgccaaaatgctggcgtagtaggtcaagactctgagactatcgaactcgcacctgaatacgctgaggctatcgcaacccgtgcaggtgcgctggctggcatctctccgatgttccaaccttgcgtagttcctcctaagccgtggactggcattactggtggtggctattgggctaacggtcgtcgtcctctggcgctggtgcgtactcacagtaagaaagcactgatgcgctacgaagacgtttacatgcctgaggtgtacaaagcgattaacattgcgcaaaacaccgcatggaaaatcaacaagaaagtcctagcggtcgccaacgtaatcaccaagtggaagcattgtccggtcgaggacatccctgcgattgagcgtgaagaactcccgatgaaaccggaagacatcgacatgaatcctgaggctctcaccgcgtggaaacgtgctgccgctgctgtgtaccgcaaggacaaggctcgcaagtctcgccgtatcagccttgagttcatgcttgagcaagccaataagtttgctaaccataaggccatctggttcccttacaacatggactggcgcggtcgtgtttacgctgtgtcaatgttcaacccgcaaggtaacgatatgaccaaaggactgcttacgctggcgaaaggtaaaccaatcggtaaggaaggttactactggctgaaaatccacggtgcaaactgtgcgggtgtcgataaggttccgttccctgagcgcatcaagttcattgaggaaaaccacgagaacatcatggcttgcgctaagtctccactggagaacacttggtgggctgagcaagattctccgttctgcttccttgcgttctgctttgagtacgctggggtacagcaccacggcctgagctataactgctcccttccgctggcgtttgacgggtcttgctctggcatccagcacttctccgcgatgctccgagatgaggtaggtggtcgcgcggttaacttgcttcctagtgaaaccgttcaggacatctacgggattgttgctaagaaagtcaacgagattctacaagcagacgcaatcaatgggaccgataacgaagtagttaccgtgaccgatgagaacactggtgaaatctctgagaaagtcaagctgggcactaaggcactggctggtcaatggctggcttacggtgttactcgcagtgtgactaagcgttcagtcatgacgctggcttacgggtccaaagagttcggcttccgtcaacaagtgctggaagataccattcagccagctattgattccggcaagggtctgatgttcactcagccgaatcaggctgctggatacatggctaagctgatttgggaatctgtgagcgtgacggtggtagctgcggttgaagcaatgaactggcttaagtctgctgctaagctgctggctgctgaggtcaaagataagaagactggagagattcttcgcaagcgttgcgctgtgcattgggtaactcctgatggtttccctgtgtggcaggaatacaagaagcctattcagacgcgcttgaacctgatgttcctcggtcagttccgcttacagcctaccattaacaccaacaaagatagcgagattgatgcacacaaacaggagtctggtatcgctcctaactttgtacacagccaagacggtagccaccttcgtaagactgtagtgtgggcacacgagaagtacggaatcgaatcttttgcactgattcacgactccttcggtaccattccggctgacgctgcgaacctgttcaaagcagtgcgcgaaactatggttgacacatatgagtcttgtgatgtactggctgatttctacgaccagttcgctgaccagttgcacgagtctcaattggacaaaatgccagcacttccggctaaaggtaacttgaacctccgtgacatcttagagtcggacttcgcgttcgcgtaa |
| *pTlpA* | gctgtcaccggatgtgctttccggtctgatgagtccgtgaggacgaaacagc |

Table S3 Primers used to construct plasmids of Phe metabolic pathway

| **Gene** | **Primer** | **Sequence (5’-3’)** |
| --- | --- | --- |
| ***stlA*** | pM1s3AsR_TS-C7-*stlA*-F | CACCACAGCCAGGATCCAATGAAAGCGAAAGATGTTCAGCCG |
|  | pM1s3AsR_TS-C7-*stlA*-R | CTCGCCCTTGCTCACCATTTCTTCCAGCATGATTTCCGGC |
| ***phep*** | pM1s3AsR_TS-C7-*phep*-F | AGCGCACGAGGGAGCTTCCAGGGGGAAACGCCTGG |
|  | pM1s3AsR_TS-C7-*phep*-R | ACCAGGCGTTTCCCCCTGGAAGCTCCCTCGTGCGC |
| ***pma*** | pACYCDuet-*pma*-F | CATATGAACATCAGCCGTCGTAAATTGC |
|  | pACYCDuet-*pma*-R | CTCGAGCTTCTTGAAACGGTCC |
| **T7 RNA ply** | pGEX-4T-2-T7 RNA ply-F | ACTCTTCCTTTTTCAATATTATTGAAGCA |
|  | pGEX-4T-2-T7 RNA ply-R | CTGTCAGACCAAGTTTACTCATATAT |
| ***kanR*** | pGEX-4T-2-T7 RNA ply-*kan*-F | tattgaaaaaggaagagtatgattgaacaagatggattgcacgc |
|  | pGEX-4T-2-T7 RNA ply-*kan*-R | gtaaacttggtctgacagtcagaagaactcgtcaagaaggcg |
